# Water-Soluble Electrospun Nanofibers as a Method for On-Chip Reagent Storage

**DOI:** 10.3390/bios2040388

**Published:** 2012-10-08

**Authors:** Minhui Dai, Shengquan Jin, Sam R. Nugen

**Affiliations:** 1 University of Massachusetts, 102 Holdsworth Way, Amherst, MA 01003, USA; E-Mails: mdai@foodsci.umass.edu (M.D.); haiquanjsq@msn.com (S.J.); 2 Lab of Biosystems and Microanalysis, State Key Laboratory of Bioreactor Engineering, East China University of Science & Technology, Shanghai 200237, China

**Keywords:** polyvinylpyrrolidone (PVP), nanofiber, electrospinning, microfluidic biosensor, enzyme storage

## Abstract

This work demonstrates the ability to electrospin reagents into water-soluble nanofibers resulting in a stable on-chip enzyme storage format. Polyvinylpyrrolidone (PVP) nanofibers were spun with incorporation of the enzyme horseradish peroxidase (HRP). Scanning electron microscopy (SEM) of the spun nanofibers was used to confirm the non-woven structure which had an average diameter of 155 ± 34 nm. The HRP containing fibers were tested for their change in activity following electrospinning and during storage. A colorimetric assay was used to characterize the activity of HRP by reaction with the nanofiber mats in a microtiter plate and monitoring the change in absorption over time. Immediately following electrospinning, the activity peak for the HRP decreased by approximately 20%. After a storage study over 280 days, 40% of the activity remained. In addition to activity, the fibers were observed to solubilize in the microfluidic chamber. The chromogenic 3,3′,5,5′-tetramethylbenzidine solution reacted immediately with the fibers as they passed through a microfluidic channel. The ability to store enzymes and other reagents on-chip in a rapidly dispersible format could reduce the assay steps required of an operator to perform.

## 1. Introduction

Lab-on-a-chip (LOC) devices have been applied in many fields, such as point-of-care diagnostics, bio-warfare detection and food safety. However, their application as biosensors often requires the use of sensitive bio-reagents to enable detection [1]. Frequently, reagents are pumped into the device from an off-chip source using a syringe pump [2] or pneumatic pump [3]. Unfortunately, incorporation of these peripherals reduces the portability and miniaturization of a finished device. Ideally, a LOC would store all necessary reagents directly on-chip thereby reducing user handling and simplifying the final device. 

Nanofibers containing multiple components are a promising method for on-chip storage [4]. Electrospinning is not only a simple, inexpensive and versatile process to form nano-scale fibers with large surface areas [5] but also a rapid way to evaporate solvent while maintaining the integrity of the components [6]. These unique features ensure the potential applications of electrospun bio-composite nanofibers in many features, such as clothing [7,8], membrane distillation [9,10], biomedical sensing [11], catalysis [12], biomedical application [13] and enzyme storage [14]. 

Polyvinylpyrrolidone (PVP), a common hydrophilic polymer [15], has good film formation properties which makes it popular for electrospun nanofibers. PVP is soluble in water and absorbs up to 40% of its weight at ambient conditions [16]. Typically, enzymes have a shortened shelf life when stored at ambient conditions and require lyophilization. Electrospinning, which is able to dehydrate samples in a timescale of milliseconds, may present an ideal alternative preservation method for biological samples [17]. In this study, PVP electrospun nanofibers were made to store HRP under ambient conditions. The fibers serve as a mechanism to not only store the enzyme, but also to distribute it evenly within the sample solution. The solubility and small dimensions of the fibers make them ideal for rapid delivery of reagents.

## 2. Experimental Section

### 2.1. Materials

PVP of M.W. 1,300,000 was purchased from Sigma-Aldrich (St. Louis, MO, USA). D(+)-Sucrose (>99%), horseradish peroxidase (HRP) and a 1-step slow 3,3′,5,5′-tetramethylbenzidine (TMB) kit (1-Step^TM^Slow TMB-ELISA) were obtained from Thermo Fisher Scientific Inc. (Rockford, IL, USA). 

### 2.2. Electrospun Nanofiber Preparation

The spinning solutions were prepared by mixing 15 wt% PVP, 5 wt% sucrose and 0.01 mg/mL HRP/aqueous solution. The samples were stirred gently for 30 min to allow for a uniform distribution. The mixed solutions were drawn into a 1 mL plastic syringe (National Scientific Company, Rockwood, TN. USA), equipped with a stainless steel 22 gauge blunt needle (SmallParts, Inc., Seattle, WA, USA). The positive electrode from a high voltage (10–30 kV) DC power supply (Gamma High Voltage Research Inc., Ormond Beach, FL, USA) was clipped on the needle. A grounded copper plate used as a collector was placed 12 cm away from the tip of the needle. The nanofibers were formed using a potential of 20 kV and pumped at 100 μL/h using a syringe pump. The collected fibers were removed from the copper plate and placed in a desiccator at room temperature until use.

### 2.3. Scanning Electronic Microscope (SEM)

Fiber mats were sputter-coated with gold for 90 s and observed with a scanning electronic microscope (JEOL JSM 6320F) at an accelerating voltage of 5 kV. The average fiber diameters and the standard deviations were calculated from the SEM images using the software ImageJ (National Institutes of Health) to measure a total of 30 fibers.

### 2.4. HRP Activity Measurement

Fibers mats were removed from the copper plate and cut into round pieces using a 1 cm diameter punch. The masses of the fiber pieces were extremely small and consequently five cut pieces were then cumulatively weighed and an average mass was determined. From the average mass, the quantity of HRP within each cut piece was calculated using the original mass fraction of the dry constituents. Each piece was placed into a single well of a 96-microtiter plate for reaction analysis. The enzyme activity was compared to an equal mass of HRP which had not undergone the electrospinning process in order to compare the change in activity. The indicator TMB is a chromogen that yields a blue color when oxidized, typically as a result of oxygen radicals produced by the hydrolysis of hydrogen peroxide by HRP. The oxidized TMB has a maximal absorbance at 652 nm [18]. For the activity assay, 150 μL of a 1-step slow TMB kit was added to 100 μL water and added to the well containing the nanofibers. The plate was then inserted into a microtiter plate reader (Biotek, Winooski, VT, USA) where the absorption at 652 nm was measured over time. Activity was quantified by an increase in adsorption at 652 nm. A negative control representing equivalent concentrations of PVP and sucrose without HRP was also measured. The enzyme activity comparison was measured immediately after electrospinning to determine the initial activity. After storage in a desiccator for 45 and 280 days, enzyme activity within the fibers was again characterized.

### 2.5. On-Chip Microfluidic Devices with Nanofibers

In order to demonstrate the ability of enzyme containing nanofibers to deliver reactive enzymes on-chip, a microfluidic on-chip device was designed using autoCAD and fabricated by a 30 W desktop laser (Epilog Laser, Golden, CO, USA). The device consisted of two bonded pieces of PMMA, the nanofiber mat and an absorbent pad. One of the pieces of PMMA was structured with microfluidic channels, an inlet port and a cavity for the absorbent pad and nanofiber, while the other piece remained unpatterned. These structures were all fabricated on the PMMA sheet using laser ablation. Following laser ablation, polymethyl methacrylate (PMMA) chips were sonicated in 15% isopropanol for 5 min (Branson Ultrasonic Corp, Danbury, CT, USA) and given UV treatment for 5 min. To bond the two PMMA pieces, 20 μL 2,4-pentanedione was deposited onto the unpatterned piece of PMMA and allowed to rest for 25 s before the PMMA was spun at 1,250 rpm on a spin coater for 5 s (Laurell, North Wales, PA, USA) [19]. The nanofiber mats and an absorbent pad (CF5, Whatman, UK) were placed into their respective laser ablated chambers of the patterned PMMA and the two PMMA pieces were then pressed together at 4,500 MPa at 37 °C for five minutes using a hydraulic press with heated platens (Carver Inc., Wabash, IN, USA). 

In order to qualitatively demonstrate the activity of the enzymes in the microfluidic chamber, 100 μL of the TMB solution was placed into the inlet of the microfluidic chip. The solution was transported through the channels and into the nanofiber chamber using capillary flow. Once in the chamber, the solution dissolved the nanofibers and continued onto the absorbent pad. The change in color was observed visually and captured with a camera.

## 3. Results and Discussion

### 3.1. Morphology of Nanofibers

**Figure 1:**
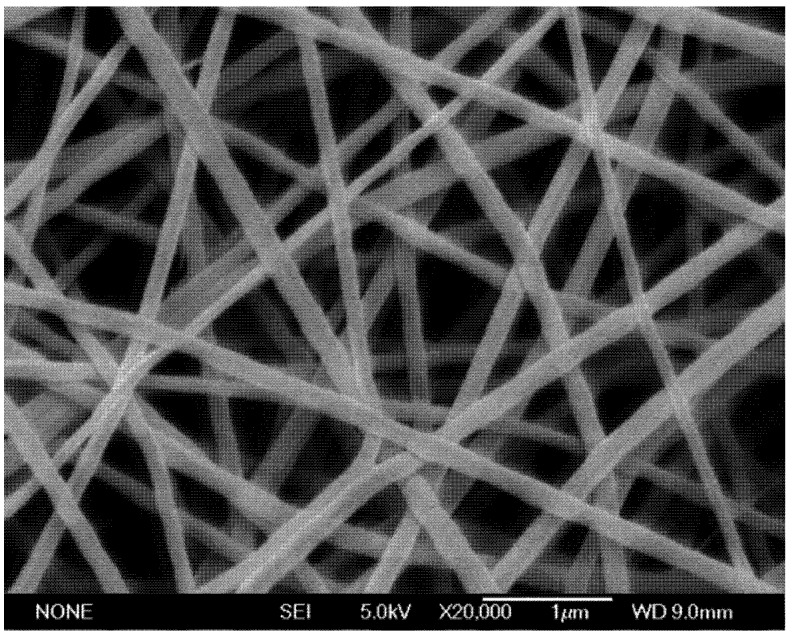
The scanning electron microscopy (SEM) image of polyvinylpyrrolidone (PVP) nanofibers electrospun from 15 wt% PVP, 5 wt% wt sucrose in 0.1mg/mL horseradish peroxidase (HRP) water solution at a gap distance of 12cm with an applied voltage of 20kV.

Following electrospinning for 1 h, nanofiber mats of approximately 2 cm diameter were removed from the copper collection plate. The individual PVP fibers containing sucrose and HRP had an average diameter of 155 ± 34 nm (Figure 1). The addition of up to 10% (wt/v) sucrose and 1% (wt/v) protein did not have an effect on the morphology of the nanofibers.

### 3.2. HRP Enzyme Activity

HRP activity was determined by reacting with TMB. At the onset of this reaction, the oxidized TMB produces a blue color with an absorbance peak at 652 nm. A continuation of the reaction yields a yellow shift with an absorbance peak at 450 nm and a subsequent drop at 652 nm. 

**Figure 2:**
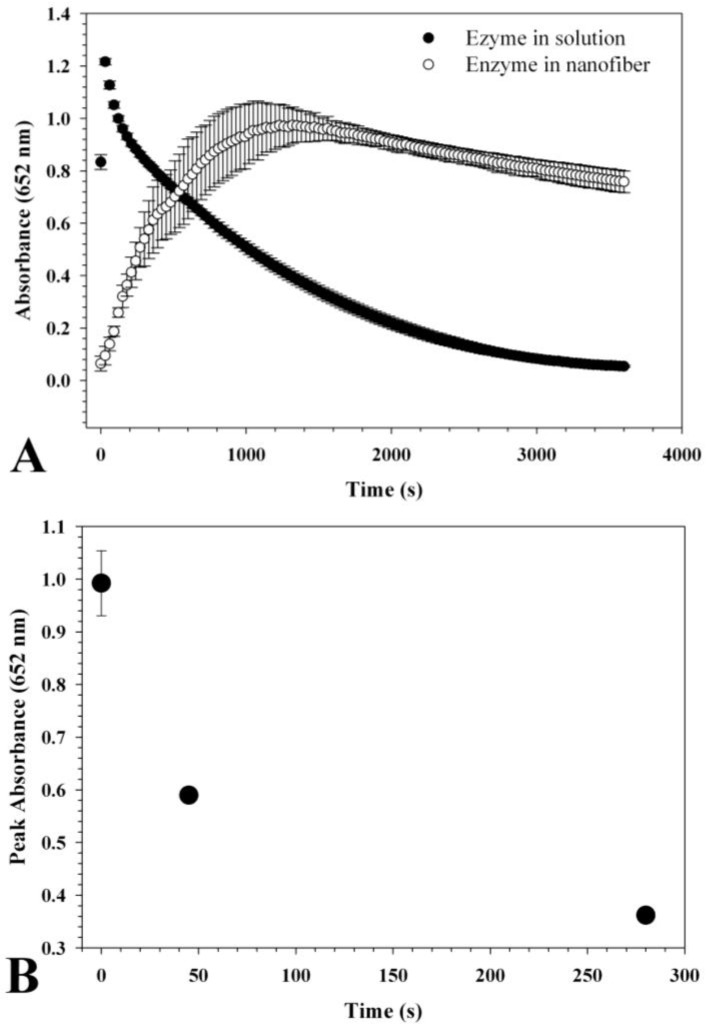
(**A**) Horseradish peroxidase (HRP) activity before and after electrospinning was detected by a 1-step slow TMB kit. 150μL 1-step slow TMB kit mixed with 100μL water was used by measuring the change in absorption at 652nm every 15 s for 1 h. The equivalent quantity of HRP was 0.04 μg. The reaction initially oxidized the TMB substrate yielding a blue color at 652 nm peak in absorbance. As the reaction progressed, the color shifted to yellow and had maximal absorbance at 450 nm. (**B**) The rate of inactivation was rapid at first and slowed over time. After 280 days, the enzyme activity was approximately 40%.

The average weight of each 1 cm fiber mat was 0.8 mg and was subsequently calculated to contain approximately 0.04 μg HRP. Therefore, during activity measurements, the control contained 0.04 μg HRP with similar ratios of PVP and sucrose. Following the addition of TMB-containing reaction solution to the nanofibers, the absorbance at 652 nm was monitored over time Figure 2(A). The results indicate that the HRP control solution without having been electrospun peaked at 30 s and then sharply declined. The average maximum absorption for the control was 1.21. The absorption peak of the electrospun nanofibers was 0.99 which occurred only after 1,300 s. This is most likely due to slower hydration in the presence of the PVP matrix. Part of the slow hydration may have been caused by bunching of the mat which had a relatively large area being placed into a relatively small well. Following desiccated storage at room temperature for 45 days, the activity of the electrospun HRP decreased to 60% and decreased further to 40% after 280 days. The rate at which the enzyme lost activity appeared to decrease over time suggesting possible stabilization (Figure 2(B)). 

### 3.3. On-Chip Microfluidic System

**Figure 3:**
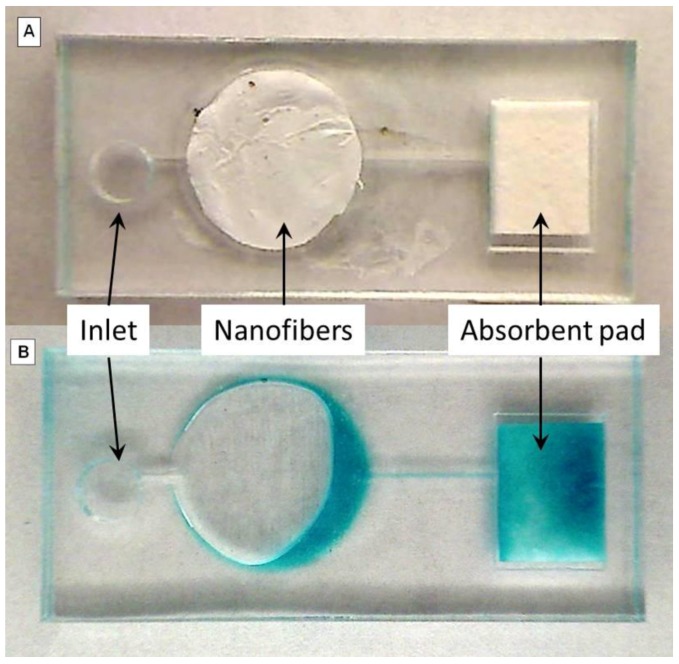
Images of electrospun nanofiber in microfluidic chip. The image demonstrates how a nanofiber mat can be incorporated into a microfluidic chip. The images are before (**A**) and after (**B**) 100μL of a 1-step TMB reaction solution was added. The color change was observed after approximately 60 s.

Electrospun nanofiber mats in the microfluidic chip appeared white prior to the addition of the TMB solution Figure 3(A). After the addition of the TMB solution, the fiber dissolved and became transparent soon after the solution reached the white mat. After approximately 60 s, the mat turned blue and the solution passed through the microfluidic channel onto the absorbent pad Figure 3(B). This reaction time was significantly shorter than the results observed using the plate reader. After the solution passed through the nanofiber channel and onto the absorbent pad, there were no visible signs of the nanofiber remaining thus suggesting it was fully dissolved. Microfluidic chips were tested both immediately following fabrication and after storage in a room temperature desiccator for 45 days. Given the nature of the assay, it was not possible to visually quantify the enzyme activity of the nanofibers within the microfluidic chip.

## 4. Conclusions

In these experiments, we demonstrated the use of water-soluble nanofibers for the storage of enzymes within a microfluidic chip. Horseradish peroxidase was selected due to its ubiquity in diagnostic assays. The results demonstrated an initial drop in activity following electrospinning. After 45 days the activity dropped to 60% and by 280 days was 40%. The storage was demonstrated in a microfluidic chip where the nanofibers were able to be rapidly dissolved and the released enzymes then catalyzed a reaction with TMB and hydrogen peroxide. 

Water-soluble nanofibers can provide an ideal reagent format for microfluidics. Dehydration during the electrospinning process occurs over very short periods of time resulting in an almost instantaneous transformation from an enzyme in solution to a dried enzyme trapped in a polymer and sucrose matrix. The ability to store sensitive reagents inside a microfluidic sensor l enables increased portability and user friendliness. By containing reagents within the chip, the operator will be required to perform fewer steps thus increasing the ease-of-use. A self-contained device would be ideal for resource-limited areas where such characteristics are required.
